# Know Yourself: Physical and Psychological Self-Awareness With Lifelog

**DOI:** 10.3389/fdgth.2021.676824

**Published:** 2021-08-11

**Authors:** Jiayu Li, Weizhi Ma, Min Zhang, Pengyu Wang, Yiqun Liu, Shaoping Ma

**Affiliations:** Department of Computer Science and Technology, Institute for Artificial Intelligence, Beijing National Research Center for Information Science and Technology, Tsinghua University, Beijing, China

**Keywords:** lifelog, data mining, machine laerning, sleep quality, personality, mood, depression

## Abstract

Self-awareness is an essential concept in physiology and psychology. Accurate overall self-awareness benefits the development and well being of an individual. The previous research studies on self-awareness mainly collect and analyze data in the laboratory environment through questionnaires, user study, or field research study. However, these methods are usually not real-time and unavailable for daily life applications. Therefore, we propose a new direction of utilizing lifelog for self-awareness. Lifelog records about daily activities are used for analysis, prediction, and intervention on individual physical and psychological status, which can be automatically processed in real-time. With the help of lifelog, ordinary people are able to understand their condition more precisely, get effective personal advice about health, and even discover physical and mental abnormalities at an early stage. As the first step on using lifelog for self-awareness, we learn from the traditional machine learning problems, and summarize a schema on data collection, feature extraction, label tagging, and model learning in the lifelog scenario. The schema provides a flexible and privacy-protected method for lifelog applications. Following the schema, four topics were conducted: sleep quality prediction, personality detection, mood detection and prediction, and depression detection. Experiments on real datasets show encouraging results on these topics, revealing the significant relation between daily activity records and physical and psychological self-awareness. In the end, we discuss the experiment results and limitations in detail and propose an application, *Lifelog Recorder*, for multi-dimensional self-awareness lifelog data collection.

## 1. Introduction

As an important concept in biological and psychological studies, self-awareness is the experience of own personality or individuality of an individual (1). It describes how an individual consciously understands their character, feelings, and desires. The previous studies on self-awareness mainly explored the physical and mental status of people with human effort, such as field research study (2), user study (3), and questionnaires (4). However, these methods are time-consuming and limited to the laboratory environment, which is impossible for large-scale applications in daily life.

With the development of portable devices and storage techniques, recording daily life with digital sensors, such as wristbands and smartphones, becomes increasingly popular. As a result, lifelog, as the data reflection on life experience passively gathered and processed with multimedia sensors (5), has drawn attention in academy and industry. The lifelog provides a new possibility for self-awareness detection, as it has been proved that daily activity reflects the subjective health status of people (6).

Current lifelog analysis mainly focuses on the objective reflection of lifelog for a specific short-term goal, such as scene searching, diet monitoring, and item recommendation. However, the ultimate goal of lifelog should be to record detailed memories in the daily activities of an individual and help people better understand and live their life (5). Therefore, it is necessary to move from objective analysis to subjective understanding of lifelog, and connect lifelog with the lifelogger more tightly.

Having noticed this tendency in lifelogging research studies, we propose to improve the life-long self-awareness of people on the physical and mental status with the help of lifelog records. In this study, we integrate a series of our recent research studies about applying lifelogging to detect subjective self-awareness, revealing the great potential of lifelog on self-exploring for ordinary people.

To be specific, four topics about life-long self-awareness exploration based on lifelog data are introduced, from physical analysis on **sleep quality prediction**, to psychological exploration about static **personality detection**, real-time **mood detection and prediction**, and further **depression mood detection**. Generally, experiments on all topics follow the same schema: 1) lifelog data collection, 2) activity features extraction, 3) label tagging, and 4) prediction/detection with popular machine learning models. All four topics give insight into the relation between lifelog and the physical/psychological status of lifeloggers, and the experiments show that lifelog does reflect abundant information for self-understanding. However, due to the limitation of dataset collection, cross-task analyses are not applied in these experiments. To formalize lifelog data collection and achieve the goal of long-term stable records for ordinary people, we build an application for semi automatic multi-dimensional dataset collection, which frees users from integrating heterogeneous data, helpful for the more in-depth research studies on lifelog and psychology in the future.

The main contributions of this study are as follows: 1) We reveal a new inter-disciplinary direction for lifelog and psychology. A relation from objective data to subjective self-awareness is established. Moreover, four new topics were proposed about sleep, personality, and mood. To the best of our knowledge, this is the first study to conduct these subjective understanding tasks *via* lifelog records. Traditionally, psychological experts assist people in detecting their status. With lifelog, ordinary people can make daily detection by themselves, and early awareness of physical and mental diseases (e.g., depression) is possible. 2) We summarize a schema for lifelog-based analysis, prediction, and intervention, providing a flexible and general framework for practical applications on multiple scenarios. Different from traditional machine learning problems, lifelog data are usually highly heterogeneous and personalized, based on a self-collected dataset. So the data collection, feature processing, and label tagging occupy a large chunk in the experiments. 3) Experiments on real data show encouraging results on multiple self-awareness tasks, demonstrating the capacity of lifelog for automatic perception on self-awareness. Moreover, we provide a novel light-weight method and application for collecting lifelog data. 4) Active or passive data by simple portable devices are supported in experiments. Meanwhile, the applied models are light and possible to deploy on personal devices such as smartphones. This means the data collection and analysis can be dealt with locally, which helps protect privacy for users.

In the rest of this study, we will review the related work in section 2, and give an overview of the schema and topics in section 3. Then, how we adopt the four topics about lifelog-based self-awareness are introduced, respectively, in sections 4–7. After that, in section 8, we will summarize and discuss the results and limitations of our experiments. Based on the discussions, the data collection method and application are shown in section 9. Finally, conclusions and possible future directions are present in section 10.

## 2. Related Work

### 2.1. Personalized Lifelog Research Study

With the development of portable devices, lifelog collection and storage have become convenient. Therefore, lifelog-based research study has drawn much attention, especially personalized applications.

One of the most fundamental and essential tasks for lifelog is to build search engines on the recorded data (7, 8). As lifelog includes details about personal daily life, it can serve as memory assistance, helping people recall events in the past. Various search engines are built for searching lifelog in an interactive manner (9–11), where users can scan the lifelog records and give real-time feedback to search engines. The lifelog records are also used for self-monitoring on daily activities such as diet (12), smoking cessation (13), and exercise (14). These studies utilize active or passive logging records to help users adjust behaviors. However, the self-monitoring research studies focus on the objective collection and recording of daily activities without in-depth analysis and understanding of the data. Further, some researchers pay attention to enhance traditional retrieval tasks with lifelog information. For example, Kumar et al. (15) give suggestions for activities from history timeline records in lifelog. Uno et al. (16) recommend music based on contextual phenomena and the historical preference of users. Nakamura et al. (17) transform lifelog into text and recommend similar TV programs for users.

These previous research studies either focus on the objective reflection of lifelog or design for an external short-term goal. However, they ignore that lifelog data are powerful for exploring the overall status of a recorder, especially mental status. The most distinctive feature of lifelog is that it can record information about the passively and thoroughly. Therefore, lifelog can help reflect the physical and mental status of user over a long period. It is valuable and possible to utilize lifelog for real-time and life-long self-awareness for ordinary people without professional intervention.

### 2.2. Machine Learning in Psychology

As the fastest-growing application of computer science, machine learning is widely used for various traditional scenarios, developing interesting topics in inter-disciplines (18). Psychology is one representative example. Since more and more precise sensors are used for research studies on psychology, it becomes difficult for the manual effort to deal with the large-scale data. Algorithms can help detect phenomena and features in the records, and mine the underlying information about human activity.

On one hand, machine learning algorithms are used for modeling human behavioral patterns in traditional psychological laboratory studies. One typical example is that Koelstra et al. (19) proposed a well-known dataset in emotion analysis, Deap, which includes electroencephalogram (EEG), facial expression, and other biometric data of 32 participants watching emotional music videos. Based on the dataset, a large amount of machine learning methods were proposed to improve the emotion prediction accuracy or find in-depth information about emotion and physiological feature, including support vector machine (SVM) (20, 21), convolutional neural network (CNN) (22), and residual long short-term memory (LSTM) (23). On the other hand, the development of portable devices and the internet has provided new resources for revealing the mental status of people. Machine learning, especially deep learning methods, is helpful for dealing with big data on the internet. Lin et al. (24) detected the stress level of users based on their tweet text and social relationship with the CNN model. Tweeter information is also used for suicide probability prediction with a deep learning model (25). Machine learning methods are also applied for digital personal information, such as phone use and smartphone sensor records, for stress and mood detection (26).

Compared with the laboratory studies and internet-based psychological research studies, lifelog-based research studies try to solve similar problems with entirely different information and records. Different from laboratory study data, lifelog is collected in an open environment, with complex and personalized simulations. So the lifelog records contain more noise and personal data. The lifelog-based study is also diverse from the large-scale internet research studies. Lifelog is more personalized, with more data for each user, but much fewer users in total. Therefore, more issues about privacy and individuation should be taken into consideration when designing machine learning algorithms in lifelog. There are already some research studies considering daily records and psychology, such as prediction of next mood record with history mood sequence using LSTM network (27), or filling missing data in heart rate with CNN encoder-decoder structure (28). However, they usually focused on one or two kinds of data in daily life. In this study, we try to consider the integration of multi-modal sensor data about user and environment to detect the mental status of user with machine learning methods, bridging the gap between daily records and mental health status.

## 3. Overview of Schema and Topics

**Figure 1:**
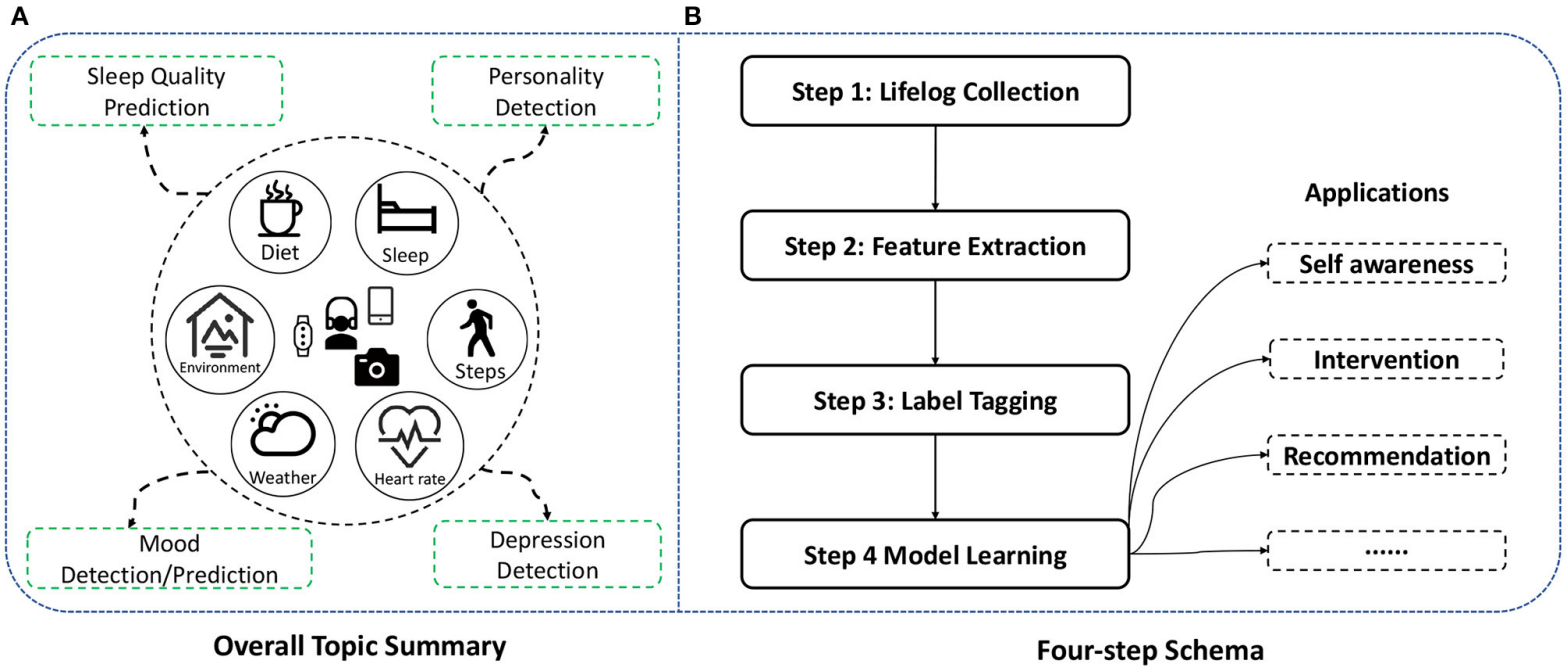
An illustration for data and topic generation and the lifelog analysis schema. **(A)** Lifelog is recorded automatically with wristbands, smartphones, and cameras and then used for the four topics. **(B)** Four-step schema is proposed for analyzing each topic, and the model can be further used for various applications, such as self-awareness, status intervention, and activity recommendation.

First, we summarize a schema for applying lifelog to understanding self-awareness, as shown in Figure 1B. The schema consists of four steps:

- **Step 1: Lifelog collection**. Since there is no widely acknowledged public dataset about lifelog, we need to collect daily activities for each experiment with portable sensors passively and actively.
- **Step 2: Feature extraction**. After removing noise and invalid data, features are extracted and calculated from the lifelog by categories such as diet, activity, biometrics, and environment.
- **Step 3: Label tagging**. Labels are usually tagged manually with participants and then used for supervised learning of prediction or detection model.
- **Step 4: Model learning**. Many machine learning models can be used for training. However, we suggest not to use too complex models, such as deep learning methods. Because of privacy concerns, it is better to make predictions and even training on local mobile terminals. So light models are preferred for our lifelog analysis schema.

Following the schema, the lifelog is first collected for each user, as shown in the middle of Figure 1. Smartphones, wristbands, and wearable cameras are used for recording data automatically. Generally, various multi-modal data are gathered, including diet records, sleep quality, activity (e.g., steps), biometrics (e.g., heart rate), context information (e.g., weather), and environment. It is worth noting that not all types of data are compulsory for each task. Actually, the selection of records and features is quite flexible. According to the experiment condition or existing dataset, one or more categories of features can be used for experiments.

Based on the dataset collection, four topics are selected to explore the life-long physical and psychological self-awareness with lifelog. We start from the physical understanding, **sleep quality prediction** from lifelog. Instead of traditional monitoring methods during sleep, we study how to predict sleep quality in advance based on daytime activities. Then, we move to the psychological scenario, detecting the ability for **personality detection** of people with daily life records, which provides a time-saving method for predicting Big-Five personality. A more in-depth study on psychological application pays attention to real-time **mood detection and prediction**. Unlike stable personality, the mood is more flexible, so the prediction is more challenging. The mood detection and prediction are also quite meaningful, as mood awareness is essential for mental health. Finally, we take a further step about mood status, conducting **depression detection** for patients and healthy people. The abnormal mood detection task makes real-time mental intervention possible in daily life.

Life-long detection and intervention of self status are the focus for all four topics. By experiments on real datasets, we show that ordinary people can gain physical and mental self-awareness more fully and deeply with the help of lifelog.

With these self-awareness tasks based on lifelog, there can be many exciting and essential applications. When an explainable system for sleep quality prediction is conducted, the system can give suggestions at bedtime if poor sleep is predicted, such as drinking milk if the user is too nervous or doing some sports if the user lacks exercise at day. Based on real-time mood detection, the system can intervene when an abnormal mood is detected. For instance, it can play some peaceful music for the user or show suggestions about mood adjustment. If the user tends to self-harm or suicide, the system may also help contact family and doctors. These applications all focus on long-period detection of users and provide life-long benefits.

In experiments, the cold-start problem is especially considered. Information of some users is shown only in the test phase for models to evaluate the capacity of the system to deal with new users.

When analyzing the experiment results, we find it difficult to conduct cross-task comparison due to inconsistent dataset collection. Since lifelog collection is onerous work, it is hard to collect overall multi-dimensional data at a time. In order to free the lifeloggers from annoying work of labeling and integrating data, we provide an application *Lifelog Recorder*, and application (APP)-centered lifelog collection method, which is helpful for long-term data collection for non-professional users.

## 4. Sleep Quality Prediction

### 4.1. Background and Task Definition

First, we propose the sleep quality prediction task, a physical analysis of lifelog to predict daily sleep quality with the lifelog in about 12 h before sleep.

With the help of portable devices, we are able to measure sleep quality in real time. For instance, cardiotachometer and actigraphy are used in commercial smart wristbands to record heart rate and acceleration while users are sleeping. Based on the data, the analysis algorithm can calculate energy expenditure, sleep onset time, sleep efficiency, and others, and assess an overall score to reflect sleep quality.

High-quality sleep has positive effects on mood, productivity, and mental health and, therefore, is essential for daily life (29). Some researchers paid attention to monitoring physical status based on data during sleep (30). However, on the one hand, intervention is usually not available if low sleep quality is detected while the user is sleeping. On the other hand, psychological studies show that activities during the daytime have a significant relationship with sleep quality (31). Therefore, we investigate how to predict sleep quality in advance with daytime lifelog, so that intervention can be provided before sleep.

For user *u* at day *d*_*t*_, given daytime lifelog records *l*_*t*_ at *d*_*t*_, and historical sleep quality $\vec{s}=\left[s_{0},s_{1},\ldots ,s_{t-1}\right]$ (recorded with sleep monitor or user questionnaires in terms of score/rating.), we try to extract features ${\vec{f}}_{t}$ and further predict his/her sleep quality score *s*_*t*_ at *d*_*t*_:


(1) $$\begin{array}{r} {\vec{f}}_{t}=F\left(l_{t},\vec{s}\right) \end{array}$$

(2) $$\begin{array}{r} s_{t}=M\left({\vec{f}}_{t}\right) \end{array}$$

where *F* and *M* represent the feature extraction process and the prediction model, respectively. *F* consists of various methods to obtain information from the multi-modal raw lifelog records.

### 4.2. Methods

In our study in the NII Testbeds and Community for Information access Research (NTCIR13) (32), we explore the sleep prediction task preliminarily. We follow the dataset and feature extraction in the study and conduct more experiments and in-depth analysis about sleep quality prediction. To be specific, we add more accurate models and try a different combination of weak classifier and ensemble models for overall prediction. We also add a discussion about the cold-start problem and feature ablation study. The new experiments are discussed in the next section. In this study, we first re describe the methods in the way of four-step schema:

1) **Lifelog collection**: Overall data collection is conducted with multiple wearable sensors, including wearable cameras, wristbands, and smartphones. Four categories of lifelog are recorded: biometrics, activities, external environment, and diet.

2) **Feature extraction**: For feature extraction process *F*, data are shift according to sleep time so that the last hour of the day is always the 24th h in order to ensure data consistency. Since activities closer to sleep time should be more important for prediction, we consider sum/average values all lifelog data in periods 23rd–24th, 19th–22nd, 11th–18th h as features. Given the lifelog records, statistic features are extracted, calculated, and min-max normalized in these three periods. Then, all features are concatenated to form the feature vector ${\vec{f}}_{t}$.

3) **Label tagging**: The label for this task is the sleep quality. It can be scores calculated with smartphones and smart wristbands or ratings manually labeled by the user. A single score/rating is tagged for one night, and the historical sleep quality sequence is also concatenated with ${\vec{f}}_{t}$ and used for prediction.

4) **Model learning**: *M* can be conducted with any classifier. To balance the accuracy and efficiency, weak classifiers (e.g., decision tree and Bayes net) with ensemble learning algorithms (such as boosting and bagging) can constitute a classifier, which is then trained by supervised learning.

### 4.3. Experimental Settings

**Dataset**. We use data of two lifeloggers from NTCIR13 (33) and five from our own data collection. We only count in the data with sleep quality labels, which consist of 31 days on user 1, 22 days on user 2–4, 14 days on user 5, 55 days on user 6, and 134 days on user 7. In all, 300 instances (i.e., days) from 7 lifeloggers are collected in total.

Lifelog in five categories are recorded: Biometrics include heart rate and active/rest/total calorie burn; activities include steps and active/rest time; external environment is represented with indoor/outdoor time, weather, temperature, sun-rise and sunset time, and weekday/weekend; diets include nutrition/calorie consumption; sleep timing is the time for sleep.

Most of the features are records from commercial portable devices directly, such as smart phone, smart watch, and wristbands. Diet information is collected with a fitness application on smart-phone, where users enter the barcodes or types of their meals and snack manually, and the application will convert them into calorie intake. The only post-process feature is the *active/rest time*, which is decided by the percentage of active calories in total calories. If active calories take up more than 50% of total calories in duration, it will be determined as active time, otherwise as rest time.

In the NTCIR13 dataset, the sleep quality score is calculated with smart wristbands and recorded as an integer *s* between 0 and 100. Since we formalize the task as a classification problem, the numeric sleep scores are grouped into four nominal categories: “Good” for *s* ∈ [80, 100], “Borderline” for *s* ∈ [60, 80], “Poor” for *s* ∈ [40, 60], and “Sleepless” for *s* ∈ [0, 40].

After feature process *F*, feature vector ${\vec{f}}_{t}$ of four categories and the historical sleep quality are extracted.

For the prediction model, some popular classifiers, such as BayesNet, locally weighted regression (LWL), C4.5 decision tree, logistic model trees (LMT), random forest (RF), and decision table (DT), are chosen as basic weak classifiers. Ensemble algorithms AdaBoost and Bagging are used to aggregate the weak classifiers.

**Evaluation metric**. Prediction accuracy is used as the metric to evaluate classifier performance.

### 4.4. Experiments and Results

We conduct experiments on overall prediction performance, cold-start user prediction, and ablation study on features. Compared with our previous publication (32), we add more algorithms for classification in overall prediction, and the cold-start and ablation study tasks are both new.

#### 4.4.1. Overall Prediction

We conduct 10-fold cross-validation on 300 instances of all seven users, to evaluate the performance of the various combinations of week classifiers and ensemble algorithm. At each fold, 10% (30 instances) of the dataset is split for the test set, and the others for the training set. Average accuracy among 10-fold is recorded as the final results.

The results of overall sleep quality prediction with lifelog are shown in Table 1. Among all the methods, the combination of the C4.5 decision tree and bagging performs the best, reaching an accuracy of 77.44%. This demonstrates that activity records in the daytime can predict sleep quality. Tree-structured weak classifiers (i.e., C4.5, LMT, RF, and DT) all have good performance, while the linear model (LWL) performs worse, which indicates the relationship between lifelog features and sleep quality may be complex non linear relation. For most classifiers, ensemble algorithms will improve performance. However, random forest (RF) is an exception since RF itself is an ensemble model.

**Table 1:** Results (in terms of accuracy) for overall sleep quality prediction of various classifiers with different ensemble algorithm.

**Weak classifier**	**Ensemble algorithm**		
	**- (%)**	**AdaBoost (%)**	**Bagging (%)**
BayesNet	69.36	75.08	73.40
LWL	52.18	57.57	53.53
C4.5	73.73	74.04	**77.44**
LMT	73.06	73.06	74.07
RF	72.05	69.69	70.03
DT	74.74	77.10	76.09

*Bold values all mean the best result(s) in the experiment. The underline value indicates the second best results*.

#### 4.4.2. Cold-Start User Prediction

Furthermore, we conduct cold-start user prediction to explore whether pre trained classifiers can be used for new users in sleep quality prediction. Moreover, we test the ability to make a prediction on a user without any history data by removing historical sleep features. At each time, we keep the instances of each user as the test set and train the models on the data of other six users.

**Figure 2:**
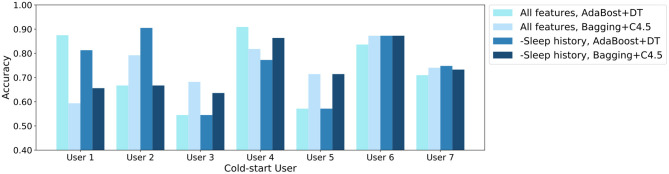
The cold-start user sleep quality prediction accuracy for seven users. The experiments are conducted with all features and features except historical sleep (*- Sleep History*), respectively.

The prediction accuracy results are shown in Figure 2. It can be observed that training with data from strangers also gains encouraging results on the sleep quality prediction for cold-start users. Moreover, although user 7 have much more instances than the other 6 users, the models trained with user 1–6 still have good performance on user 7, and the other prediction performance does not skew as well. On the one hand, it illustrates that the prediction is rather trustworthy even if we have an imbalanced dataset. On the other hand, prediction accuracy varies with users. User 4 and user 6 have a good performance, while prediction on user 3 is less accurate. This results from the personalization of lifelog, and we speculate that user 3 may have different sleep habits from others. Surprisingly, removing sleep history features results in accuracy improvement for some users. The reason might be that some users have a large fluctuation in sleep status, which introduces noise if we consider the historical sleep records.

#### 4.4.3. Ablation Study

We repeat the experiments using 10-fold cross-validation and remove one category of the feature at a time to analyze the effect of each feature category on our predictions.

As shown in Table 2, sleep timing seems to have a big share in accurate prediction, while diet data do not have a significant effect on the validation result. The small impact of diet features might be because data on calorie intake are only available for a part of user 7 lifelog. Another important point is that some of the categories contain overlapped information, such as calories burned in the biometrics category and steps in the activity category and time spent outdoors in environmental features, and time spent at home or work in the activities category. This might reduce the effect of the removal of some categories.

**Table 2:** Results (in terms of accuracy) for ablation study on different features.

**Features**	**AdaBoost+DT (%)**	**Bagging+C4.5 (%)**
Total	77.10	77.44
- Biometrics	75.44	76.11
- Activities	*77.12*	72.07
- Environment	*77.46*	74.43
- Diet	73.42	75.44
- Sleep history	73.08	72.09
- Sleep timing	**52.42**	**51.75**

*Bold values all mean the best result(s) in the experiment. The italic values mean that these ablation experiments (i.e., experiments without some features) have better results than the experiment that considers all features*.

From the above experiments, it can be concluded that lifelog at daytime can help predict the sleep quality of the user in advance, and the prediction is efficient, even for cold-start users. The prediction of sleep quality is essential in the life-long term and can help people better understand their physical status.

In the future, recommendations on activities at bedtime can be a further research topic for better sleep quality.

## 5. Personality Detection

### 5.1. Task Definition

In this section, we move from physical awareness to subjective psychological self-understanding with lifelog. This task aims to evaluate Big Five personality based on daily activities and context as the start of psychological study since personality is usually stable and essential for self-awareness.

Big Five personality (34), also known as the five-factor model (FFM), is a widely-used professional evaluation in psychology, consisting of five dimensions: Openness to Experience, Conscientiousness, Extraversion, Agreeableness, and Neuroticism. The traditional way to measure Big Five personality is by filling questionnaires, which is time-consuming and labor-intensive. Therefore, we propose a new idea to conduct personality evaluation with lifelog. In this way, a large amount of data can be collected and analyzed automatically to form Big Five personality results, available for large-scale applications.

To formalize, given lifelog records *l* in a period of time, we collect features $\vec{f}$ and predict personality scores in five dimensions $\vec{S}=\left\{s_{1},s_{2},s_{3},s_{4},s_{5}\right\}$ with a prediction model *M*.

### 5.2. Findings

We proposed the personality detection task in our previous study (35). Following the dataset processing and experiment settings in that publication, we re conduct the experiments with LR (published before) and two more models (SVM and GBDT). We also report new results about performances on each user. For completeness, we summarize the training target and then show the new results and findings. Detailed information about dataset collection and feature selection can be found in the original study (35).

**Target**. We predict each of the five dimensions in the Big Five Personality as a classification problem. To be specific, the Neuroticism-Extraversion-Openness Five-Factor Inventory (NEO-FFI) questionnaire (36) is applied to test Big Five personality as the ground truth. The output includes five dimensions of personality, of which each dimension is a score between 0 and 5, and then binarized with threshold 3.

**Figure 3:**
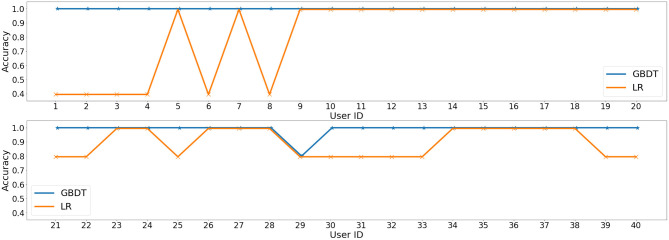
Average accuracy on five dimensions for each participant with Logistic Regression (LR) and Gradient Boosting Decision Tree (GBDT), respectively.

**Results and Main Findings**. Three traditional classifiers are used in our experiments, LR, SVM, GBDT. To evaluate the overall performance of personality prediction, we conduct five fold cross-validation and report average accuracy in Table 3. Though only six features are taken into consideration, the performance of personality prediction is surprisingly excellent, with almost all prediction results approaching 100% for SVM and GBDT. Moreover, we perform the leave-one-participant-out experiment, i.e., predict on each user, with all the others as the training set, and the average accuracy of five dimensions is shown in Figure 3. GBDT gains 100% accuracy on all users except user 29, and the simple LR also achieves 100% accuracy on more than half of the users. The promising results show that it is highly convincing to predict personality based on daily activities and habits.

**Table 3:** Personality prediction results (in terms of average accuracy of five fold cross-validation) on five dimensions of Big-Five personality.

**Dimension**	**LR (%)**	**SVM (%)**	**GBDT (%)**
Neuroticism	82.5	95.0	**97.5**
Extraversion	82.5	**100**	**100**
Openness	82.5	**100**	**100**
Agreeableness	90.0	**100**	**100**
Conscientiousness	82.5	**100**	**100**

*Bold values all mean the best result(s) in the experiment*.

**Limitations**. As the first attempt to predict personality, the present task is still quite simplified, with all personality scores binarized, and life records of all participants clear and intact. More complex processing and predicting methods should be considered in large-scale practical applications for personality detection.

## 6. Mood Detection

### 6.1. Task Definition

We consider a more complex topic in psychological self-awareness, mood detection in daily life (37). As we discuss in section 5, lifelog can be used to deduce the mental status (i.e., personality) of people. However, personality is relatively stable and predictable. However, the mood is much more dynamic and complex, so it is valuable to explore whether mood changes are detectable and even predictable in advance with daily activity records.

The mood is the background feeling that can influence the thoughts and actions of a person (38). Since mental health is getting more attention, mood detection is an essential task for both health care and self-understanding. In previous research studies, interview, survey, or self-report activities have been used for mood detection, which can be biased and inaccurate (39, 40). To capture mood changes and give real-time insights about personalized mood, we propose to detect and predict mood with automatically collected lifelog data.

For user *u*_*i*_ at day *d*_*t*_, given daytime lifelog records *l*_*t*_, we define two tasks as follow:

- **Mood detection**: Detect *u*_*i*_ mood at the wake-up time of day *d*_*t*_ in two dimensions: Valence (Stress) and Arousal (Energy).
- **Mood prediction**: Predict *u*_*i*_ mood at the wake-up time of day *d*_*t*+1_ in two dimensions: Valence (Stress) and Arousal (Energy).

### 6.2. Findings

We explore the mood detection and prediction tasks in our study in first AAAC Asian Conference on Affective Computing and Intelligent Interaction (ACII Asia 2018) (37). In this study, we list the target, main findings, and limitations of the study. Detailed information about the experiments, including dataset collection, feature extraction, and model training, can be found at Soleimaninejadian et al. (37).

**Target**. In the dataset, users choose mood labels every morning from a list of four kinds of mood, including happy, content, anxious, and depressed. Following the mood model of Thayer (41), four kinds of mood are binarized into two dimensions, negative valence and arousal, that is: *Happy*: (0,1), *Content*: (0,0), *Anxious*: (1,1), and *Depressed*: (1,0). Accurate prediction on each dimension is set as the target of prediction and detection task.

**Main findings**. In the publication in Soleimaninejadian et al. (37), traditional machine learning methods show encouraging results on both detection and prediction tasks on two-dimensional mood. The performance of mood detection demonstrates that activities in the following day can reflect mood at wake-up time. Mood prediction result indicates that activities may influence mood status the next morning. In all, the experiment shows a close and significant relationship between mood states and daily activity, and the relation can be reflected by lifelog, which confirm that it is possible to track life-long mental health with daily records and timely.

**Limitations**. In this study, mood is recorded and predicted/detected daily. However, the mood is dynamically changing with time, recording once a day is too rough for mood detection. Moreover, mood rhythms exist in both short and long term (42). Therefore, in the future, more fine-grained labels and periodic changing should be considered for mood prediction and detection.

## 7. Depression Detection and Intervention

### 7.1. Task Definition

In this section, we pay attention to detecting a kind of abnormal mood, depression, based on mood detection and prediction in the previous section.

Depression mood, as a reflection of the common psychological illness of the same name, is increasingly becoming an essential factor affecting the happiness of people. It makes people feel unpleasant and will endanger the life of the patients in severe cases (43). Therefore, in-time detection and intervention of depression mood are of great significance for mental health and life-long well-being. However, depression detection is more challenging than detection of normal mood. Since depression appears irregularly and unexpectedly (44), conventional methods like surveys and questionnaires are not able to detect the changes in time.

Lifelog is appropriate for detecting depression since it records activities automatically, and the machine learning model is fast enough to respond to unexpected depression mood. Therefore, we explore the competence of lifelog for depression detection:

Given lifelog *l* in a period of time and the corresponding series of mood records $\vec{M}=\left(m_{t_{0}},m_{t_{1}},\ldots ,m_{t_{n}}\right)$, depression detection is formulated as a classification problem: predicting *m*_*t*_*i*__ belongs to which type of mood with *l*_*t*_(*t* < *t*_*i*_) (0 ≤ *i* ≤ *n*).

### 7.2. Methods

**Figure 4:**
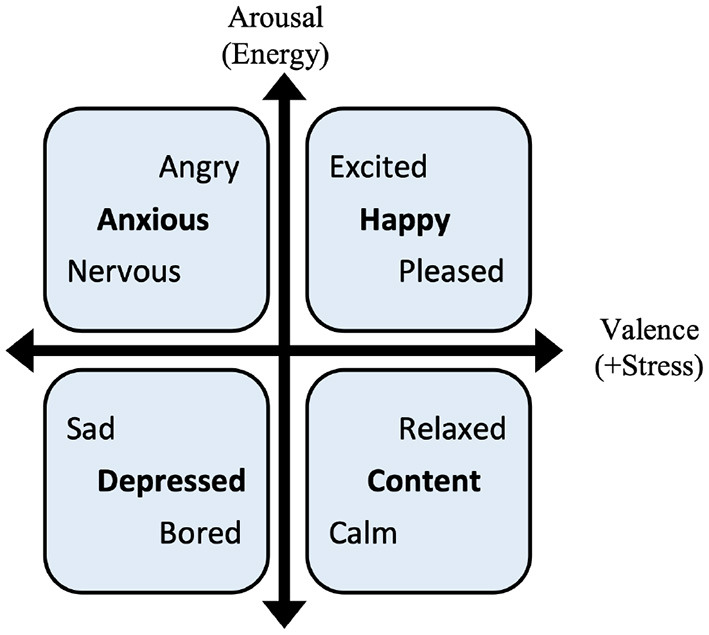
Thayer's two-dimensional mood model (32).

The depression detection task is a new task, and we have not published the results before. We describe the methods following the four-step schema for lifelog analysis:

1) **Lifelog collection**: Considering the convenience of data collection, a single wristband is used for lifelog collection, which can record activity intensity, activity type, and some primary biometrics data per minute.

2) **Feature extraction**: After removing invalid data, two groups of features are considered in data processing: activities and biometrics data. Min-max normalization is conducted on all features.

3) **Label tagging**: For mood labels, participants choose one of the 12 moods to describe their current status as shown in Table 4. Participants are asked to label their mood once per hour. The categories are set according to the mood model of Thayer, as shown in Figure 4. At the same time, participants should also record the activity they are doing. If they are engaged in multiple tasks, they only need to record the most important one. As we are interested in the intensity and valence of emotion, the 12 moods are classified into five categories (0–4), and experiments are evaluated on these categories.

**Table 4:** Twelve types of mood labels for depression detection task and their categories according to Thayer's two-dimensional mood model.

**Mood**	**Excited**	**Happy**	**Pleased**	**Annoying**	**Angry**	**Nervous**
Category	1	1	1	2	2	2
**Mood**	**Sad**	**Bored**	**Sleepy**	**Cool**	**Relaxed**	**Calm**
Category	3	3	3	4	4	0

Since mood labels are sparse, we then fill in the mood labels with two methods: 1) activity-based padding, when a user is engaged in the same activity, if the mood labels at the beginning and end of the activity are consistent, and the labels during the activity is padded the same as the beginning and end of the activity. If the labels are inconsistent at the beginning and the end of the activity, the data during the activity will be abandoned. We have to make a trade-off between label accuracy and data scale, since the mood labels are too sparse, while lifelog data are recorded per minute. 2) Label-based padding, since labels are usually recorded at the time when the mood is intense, and the change of emotion takes time, it can be considered that the labels of adjacent periods to the original mood label are consistent. In our experiment, we set the duration of adjacent periods as 2 min before and after the label.

4) **Model learning**: Same as the previous tasks, any classifier can work for depression detection. We choose two ensemble models, GBDT and random forest (RF), in experiments.

### 7.3. Experimental Settings

We collect lifelog records with wristbands from 14 healthy participants and 2 patients with depression. The patients are both clinically diagnosed, one as mild depression, and the other as moderate depression. They are well informed about the experiment. The collection includes activity type, activity intensity, steps, and heart rate, with a frequency of once a minute. During the experiment, three healthy participants dropped out. One healthy participant and one depression patient did not record any mood labels, so they are also removed from the dataset. In the end, we have 10 healthy participants and one depression patient in our data. In the process of data collection, due to the inevitable accidental occurrence of bad contact when wearing the wristbands, some of the records have missing heart rate or invalid activity intensity (labeled with 0 in the data). There are 31.3% invalid data in our collection. After removing the invalid data, the dataset contains 218,330 records during 241 days from 11 participants, varying from less than a week to more than a month per participant. The participants all report their data on consecutive days, and the concrete value of days for each user is shown in Table 5.

**Table 5:** Days of records for each user in our experiment. User 1-10 are ten healthy participants, and User 11 is the patient.

**User Id**	1	2	3	4	5	6	7	8	9	10	11
**Days**	26	23	22	17	22	25	23	23	6	2	9

*User 1–10 are 10 healthy participants, and user 11 is the patient*.

Four features in two categories are extracted: 1) Activities: activity intensity, activity type, and walking steps; 2) Biometrics: heart rate. The frequency of all the features is once per minute. Mood labels are complemented in each minute with the activity-based method and label-based method, respectively. Then, depression detection task is conducted on the 1-min features to predict the label in the minute.

For mood detection, the features within a minute are used for predicting the mood label in the minute. Since the short-term mood change is considered, the bound between detection and prediction is indistinct. In the following statement, we mix the statements of “mood detection” and “mood prediction.”

Since our lifelog dataset is not a large-scale collection, while simple classifiers are not able to achieve good performance, ensemble models are suitable for improving prediction performance. Therefore, two mainstream ensemble models, GBDT and Random Forest (RF), are applied for mood detection. In overall prediction and self-data prediction tasks, five-fold cross-validation is conducted for evaluation. As for the cold-start prediction, each user is taken as the test set to evaluate the model trained on 10 other users.

Accuracy on twelve-label classification is used as the evaluation metric.

### 7.4. Experiments and Results

#### 7.4.1. Overall Prediction

In overall prediction, five-fold cross-validation is conducted, and average accuracy is shown in Table 6. It can be concluded that GBDT performs better than RF in our experiment and that the activity-based padding method for mood labels is more accurate in mood detection. Compared with the mood detection and prediction experiments in section 6, the detection accuracy is lower in this task. There are two main reasons. On the one hand, the mood labels are more complex and dense in the depression detection task, and the available lifelog data are less, so the depression detection task is more challenging. On the other hand, our system may reveal some mood changes that users themselves have ignored, as mood tags are labeled with subjective judgment. For instance, psychological research studies have shown that about 90% of people with depression often do not realize they may be depressed in the early stages of illness (45). Therefore, our detection model may help discover some early symptoms for abnormal mood and remind users to seek professional help and early intervention.

**Table 6:** Overall results (in terms of accuracy) on depression prediction with activity-based and label-based label padding methods.

**Method**	**Activity-based padding (%)**	**Label-based padding (%)**
GBDT	**37.03**	33.49
RF	32.96	28.75

*Bold values all mean the best result(s) in the experiment*.

The prediction accuracy for each user is presented in the second column of Table 7. Prediction accuracy is quite diverse for different users. For instance, user 5 gains quite high accuracy, while the accuracy is low for user 1, 2, 3, and 7. This indicates that mood awareness is quite personalized and motivates us to conduct self-data and cold-start user detection tasks.

**Table 7:** Depression detection results (in terms of accuracy) with GBDT and activity-based padding for each user.

**User**	**Overall prediction (%)**	**Self-data prediction (%)**	**Cold-start prediction (%)**
User 1	19.89	35.77	16.65
User 2	36.10	24.84	32.74
User 3	27.81	19.89	28.06
User 4	59.73	51.08	59.33
User 5	93.26	87.78	92.85
User 6	2.21	52.49	0.0
User 7	24.37	36.00	18.8
User 8	68.11	58.56	67.85
User 9	37.78	0.0	52.00
User 10	75.21	40.68	53.35
User 11 (Patient)	55.42	51.32	56.46

#### 7.4.2. Self-Data Detection

The data of each user are used for training a specific classifier for detection. Five fold classification is conducted for each user, and average accuracy is reported in the third column of Table 7. Detection accuracy declines for users with good performance in the overall experiment, such as user 4, 5, 8, and 10. This is because less data is used for training the classifier. However, user 1, 6, and 7 have better performance in self-data prediction task. They may have strong specificity in mood patterns, which can be learned better with their own data.

We also notice that user 9 has zero accuracy in this task. This user has non-stable mood records, which is hard to be predicted with his/her data only.

#### 7.4.3. Cold-Start Detection

We further investigate the cold-start problem by setting each user as the test set and all the other 10 users as the training set. The results are shown in the fourth columns in Table 7. Cold-start results keep with overall prediction accuracy, which illustrates our classifier is suitable for new users in the depression detection task. Meanwhile, as the mood label is marked with the users, we conjecture that physical activity and mood awareness may be inconsistent for some users. The most classic example is user 6, for whom overall prediction and cold-start prediction tasks both fail, but self-data prediction gains good performance. After observing the data, we find that with almost the same activity intensity, user 6 has many labels in categories 1, while other participants stay calm or relaxed most time, which may explain the inaccuracy.

Furthermore, the cold-start prediction result (accuracy of 56.56%) of user 11 demonstrates that models trained with the data of healthy people can help predict the mood of the patients. This observation is encouraging as it reveals the similarity of mood awareness between healthy and. Therefore, we are able to design systems for patients with depression with data from healthy people or with historical data when the patient has not caught up with depression, which is of great value for practical applications.

In this task, we further investigate the relationship between psychological status and daily activity. The experiments on lifelog from healthy people and depression patients focus on more flexible and real-time detection of mood. The results show that it is possible to detect mood changes in real-time with lifelog records, demonstrating the power of lifelog on self-awareness in the psychological scenario.

It is worth mentioning that our system is a supplement and assistance for professional diagnosis, but can not replace the medical treatment by any means. In the future, deeper insight into the mechanism of emotion changes and carefully designed models for mood detection are two possible directions to improve the detection accuracy.

## 8. Discussion

Inspecting the experiments for four topics on the new direction of self-awareness with lifelog, we argue that the relation between objective lifelog data and subjective self-awareness is established efficiently. The novel direction gains insight into automatic self-tracking for life-long health and well being. We believe that it is a step toward the ultimate goal for lifelog study: to help people better understand and live their life (5).

### 8.1. Lifelog Information for Automatic Self-Awareness

In Table 8, we summarize the categories of lifelog information used in each of the four topics: sleep quality (prediction), personality (detection), mood (prediction and detection), and depression (detection). It can be observed that biometrics data (e.g., heart rate and calorie burn) are used in all topics. Activity records, environment data, and mood records are used in most topics. These categories are exactly the main focus of lifelog at present, which can be captured with state-of-the-art wearable sensors easily.

**Table 8:** A summary of lifelog information categories used in each of the four topics.

**Topic**	**Biometrics**	**Activities**	**Environment**	**Diet**	**Sleep**	**Mood**	**Demographics**
Sleep quality	✓	✓	✓	✓	✓		
Personality	✓		✓			✓	✓
Mood	✓	✓	✓	✓	✓	✓	
Depression	✓	✓				✓	

*The information is abundant and multi-modal*.

On the one hand, as we mentioned, the choice for categories is quite flexible, which means that the category combination shown in Table 8 is not compulsive for each topic but can adjust with the change of devices and record form.

On the other hand, the unused information is not meaningless for the tasks. For instance, daily activities can reflect some characteristics of personality. Sleep quality is definitely influenced by mood status. Due to the limitation of dataset collection design, we are not able to include all multi-dimensional data in our experiments. More in-depth research studies and analyses about the heterogeneous information are left for further analysis. We propose a method and application for collecting information in section 9, providing a paradigm of lifelog collection.

### 8.2. Direct and Indirect Information Extraction and Application

Both direct and indirect information are extracted from the multi-modal lifelog records. For instance, the environment category in sleep quality prediction and mood detection and prediction topics includes direct records such as weather and indoor/outdoor time, which can be captured easily. Besides, indirect features of room tidiness and decoration are contained in the external environment for personality detection topic based on reprocessing of raw image data.

The extraction process for both direct and indirect information indicates that lifelog contains abundant information for user status of great potential to explore in the future. Besides categories mentioned in Table 8, more detailed features are possible to deploy in the lifelog research studies, such as micro-expression, computer usage, and smartphone usage.

Moreover, the lifelog feature extraction provides new tasks for traditional and inter disciplinary research studies. For example, in the personality detection topic, room tidiness level and decoration level are used as features, which are extracted from photographs taken by the user. However, the requirement of tidiness and decoration index can not be satisfied with traditional object detection task or image sentiment task in computer vision scenario but needs more knowledge about psychology. Therefore, it provides new challenges and opportunities for computer vision research studies.

### 8.3. Relationship Among Tasks

**Figure 5:**
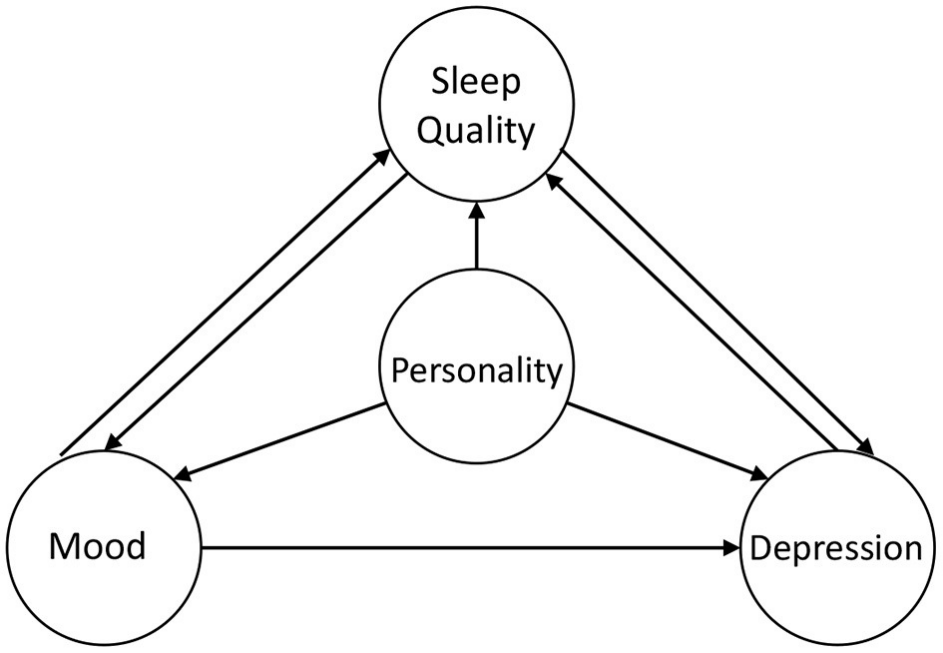
An illustration of the relationship between the four topics involved in this study.

The encouraging results for experiments show a significant relationship between lifelog records and physical and mental status. Moreover, there are relations between different tasks, as shown in Figure 5. As the most stable concept, personality provides the base for mood and the possibility of depression of an individual (46). Personality also has an influence on sleep quality. For instance, people with high neuroticism may have more problems falling asleep. Moreover, long-term bad mood or tension will cause depression, so mood detection can help discover depression at an early stage. Apparently, the daytime mood has the influence on sleep quality. On the one hand, feeling too excited or too sad will both lead to low sleep quality. On the other hand, having a good sleep helps people more energetic, so sleep quality is also important for mood detection. A similar relation also exists between depression and sleep quality.

Because the dataset collection and experiments are conducted in a progressive way, the participants of each task differ from each other, and thus, it is difficult to analyse the overall cross-task status of an individual. For future studies, it is of great value to integrate all self-awareness tasks into a framework. Joint learning methods are possible to conduct to leverage multi-task prediction accuracy. With this framework, we hope to provide an overview of the wellbeing of users and find cross-task insight into the status of the user.

### 8.4. Data Scale

Dataset collection is an important but difficult task in lifelog research study, as it includes long-term participant engagement, and multi-dimensional data should be collected at the same time. Since data recording is volunteer and users can quit the experiment at any point, data imbalance a cross dimensions and users is common in lifelog records. In the above experiments, we also suffer from data imbalance problems. From the cold-start experiments, we verify that model transfer between users is available. However, more in-depth analysis is required for data scale and data diversity. For example, we want to know how much data are enough for each task and cross-task analysis, the performance of our methods for short-term and long-term data in each task. Moreover, the consistency among tasks and participants is also an interesting problem, for instance, whether sleep quality prediction is more accurate for participants with higher mood prediction accuracy. Analysis and discussions like these are important for the practical user of lifelog research studies.

In the future, we will collect more data soundly in long term to support in-depth analysis.

### 8.5. Privacy Issues

In lifelog-related research studies, privacy is always an essential problem (5), as the lifelog collection is usually all-day and much personal information is collected during the process, such as the Global Positioning System (GPS) location and personal photographs. In our schema, a new perspective is provided for protecting privacy in lifelog analysis. The light-training models used in these tasks can be deployed on personal terminals, which means the lifelog data can be processed locally without uploading to the cloud or sharing with others. It solves the privacy problem fundamentally.

Our cold-start experiments on sleep quality detection, personality detection, and depression detection show that most methods work well for new users. Therefore, a well-trained model can be deployed directly for new users. If the user is not willing to record his/her own information, he/she can choose to use the general model in the application.

Under these settings, training and updating the models at a personal terminal are essential for higher accuracy and better user experience. Moreover, user feedback should also be considered in the interactive dynamic update of the framework.

Moreover, participants in our experiments are all informed of the details of what data we collect and how we will use the data.

### 8.6. Limitations

As the first step to link lifelog and psychology, we take some preliminary attempts in experiments. In this study, we discuss some limitations in our work.

Conventional feature extraction strategies and machine learning models are used in the experiments. Since our main purpose is to explore the possibility of reflecting psychological status from daily life records, we keep the applied strategies simple. It is helpful for calculation on the personal terminals as we argued in the privacy discussions. But some distinctive features of lifelog are ignored, such as sequential patterns, time-related features, and context information between labels. We believe including more features, as well as designing domain-specific models to deal with the lifelog features, can improve the performance of the tasks.

In this study, we review a series of progressive experiments, which are conducted on different datasets over years. As we are exploring the lifelog research scenario, the collected information is different between tasks, as shown in Table 8. Therefore, we admit that the interesting and meaningful topic of cross-task comparison and analysis on the same participants is unavailable in our study. In order to standardize the lifelog collection in the future research studies, we propose a method and application for collecting multi-dimensional data as follows.

## 9. Lifelog Recorder for Multi-Dimensional Self-Awareness Data Collection

As we discussed in the last section, the previous experiments on self-awareness were conducted on different participants with multi-dimensional information (as shown in Table 8). This experiment settings makes it difficult to evaluate overall self-awareness for one particular user. However, collecting multi-dimensional lifelog dataset is a time-consuming and laborious task. The commercially available applications are not suitable for collecting data from multiple sources (e.g., wristbands, smart-phone, and manual input) nor do most of them support the export of original data. The previous lifelog collection process for research studies highly depended on the participants, and the lifeloggers needed to collect, integrate, and upload multi-dimensional data themselves, which include heavy tasks and professional operations during the period. This process makes it hard to conduct experiments on many users for a long period. Therefore, it is necessary to develop a new application customized for psychology-related lifelog research studies.

**Figure 6:**
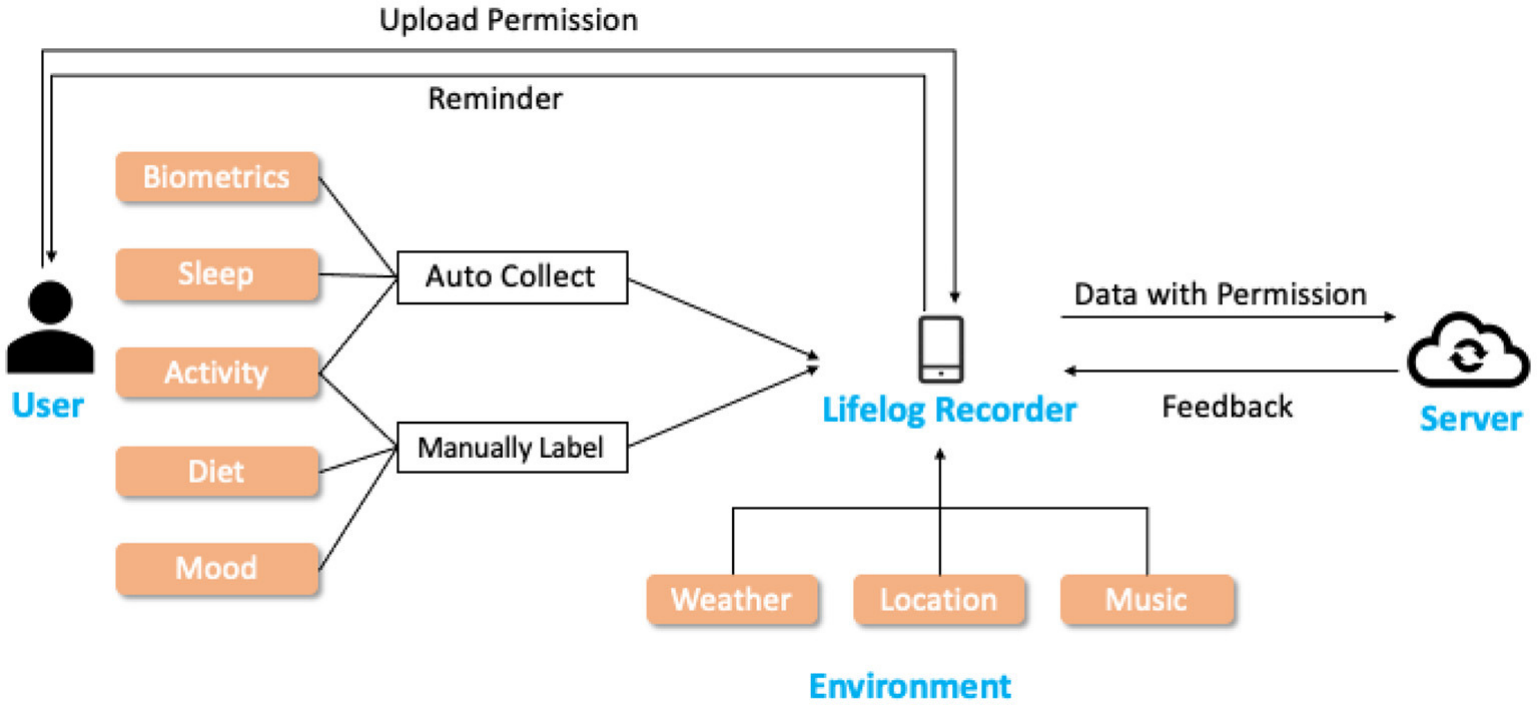
Data flow for *Lifelog Recorder* application. The application takes on the role of collecting lifelog and exchanging data with the server, which ease the burden of users.

Following the four-step schema, we develop a new APP in the Android environment, *Lifelog Recorder*, for collecting heterogeneous information as shown in Table 8. The data flow for *Lifelog Recorder* is shown in Figure 6. Centered at the *Lifelog Recorder*, lifelog data about the *user* and *environment* are collected, and uploaded to the *Server*.

During the data collection experiment, we will provide smart wrist bands for the user, and the *Lifelog Recorder* needed to be installed on the smartphone of the user. The biometrics (heart rate and intensity), sleep quality, and activities (activity type and steps) are collected with wrist-band and uploaded to *Lifelog Recorder* automatically. The user is not only asked to record their mood and activity on a regular basis but also take photographs of his/her diets. To further free the users, notifications can be customized for each user to remind them to record mood and diet in time. Environmental information, such as weather, music, and location, are recorded by the APP. In the end, with the permission of the user, *Lifelog Recorder* communicates with the server, uploads lifelog records, and is ready to receive feedback data for extended tasks such as music or news recommendation.

With the help of *Lifelog Recorder*, we are able to collect multi-dimensional data in an easier way, moving from traditional user-centered methods to an APP-centered collection method, and free the user from the onerous task of annotating and collecting lifelog. Therefore, besides researchers, non professional participants can be involved in long-period data collection tasks, which can enlarge the lifelog dataset significantly. Actually, we are collecting multi-dimensional self-awareness data in a field study with the *Lifelog Recorder*, which include all information categories in Table 8. The data will be made publically available after we finish collecting and removing the sensible personalized information.

Based on the data, we are able to explore interesting problems, such as cross-task performance.

## 10. Conclusions and Future Studies

In this study, we propose a new direction of investigating the capacity of lifelog records for tracking physical and psychological self-awareness in the life-long term. We summarize a four-step paradigm for lifelog analysis and application, including lifelog collection, feature extraction, label tagging, and model selection. Following the paradigm, four topics are proposed for insight into physical and psychological health: sleep quality prediction, personality detection, mood detection and prediction, and depression mood detection. Among the topics, some tasks based on short-term lifelog, while others need longer periods of records. But they all focus on life-long analysis and understanding of people. Experiments on real-world data show encouraging results on all tasks, revealing the great potential of lifelog for long-term self-tracking. On discussing the concerned issues and limitations of our experiments, we further put forward a method and an application for lifelog data collection.

In conclusion, we reveal a new direction of employing objective lifelog data for subjective self-awareness detection. The experiments show that lifelog is a good reflection of the physical and mental status of people. Analysis, detection, and prediction with lifelog records can help people better understand their status.

In the end, we present some directions for further study. First, prediction and detection are just the primary understanding for well being. Based on the accurate prediction, recommendation and intervention methods can be further explored. Second, as we mentioned, the light-training framework can be deployed on personal devices. Therefore, local update of the models is important in application. Moreover, we will try domain-specific features and new methods for improving the task performance. Finally, based on the *Lifelog Recorder* application, we will design an overall system to integrate all tasks automatically, and conduct cross-task in-depth analysis about task performance, data scale, and model feasibility.

## Data Availability Statement

The datasets presented in this article are not readily available because The dataset contains lifelog records of participants, such as photos and location information. Due to privacy concern, the dataset can not be made publicly accessable. Requests to access the datasets should be directed to Min Zhang, z-m@tsinghua.edu.cn.

## Ethics Statement

The studies involving human participants were reviewed and approved by Jing Qian, Tsinghua University. The patients/participants provided their written informed consent to participate in this study. Written informed consent was obtained from the individual(s) for the publication of any potentially identifiable images or data included in this article.

## Author Contributions

JL contributed to data analysis and writing and editing of the manuscript. WM contributed to the design of methods. MZ contributed to guidance and method design through the research study. PW contributed to the data collection and experiments on depression detection topic. YL and SM contributed to the discussion in the experiment and manuscript writing. All authors contributed to the article and approved the submitted version.

## Conflict of Interest

The authors declare that the research was conducted in the absence of any commercial or financial relationships that could be construed as a potential conflict of interest.

## Publisher's Note

All claims expressed in this article are solely those of the authors and do not necessarily represent those of their affiliated organizations, or those of the publisher, the editors and the reviewers. Any product that may be evaluated in this article, or claim that may be made by its manufacturer, is not guaranteed or endorsed by the publisher.

## References

[1] Duval S Wicklund RA. A Theory of Objective Self Awareness. Academic Press (1972).

[2] Rochat P. Five levels of self-awareness as they unfold early in life. Conscious Cogn. (2003) 12:717–31. 10.1016/S1053-8100(03)00081-3 14656513

[3] Lou H Changeux JP Rosenstand A. Towards a cognitive neuroscience of self-awareness. Neurosci Biobeh Rev. (2017) 83:765–73. 10.1016/j.neubiorev.2016.04.004 27079562

[4] Killian KD. Development and validation of the emotional Self-awareness questionnaire: a measure of emotional intelligence. J Marital Family Ther. (2012) 38:502–14. 10.1111/j.1752-0606.2011.00233.x 22804468

[5] Gurrin C Smeaton AF Doherty AR. Lifelogging: personal big data. Found Trends Inf Retrieval. (2014) 8:1–125. 10.1561/1500000033

[6] Bouchard C Blair SN Haskell WL. Physical activity and health. Hum Kinet. (2012) 3–20. 10.5040/9781492595717

[7] Gurrin C Le TK Ninh VT Dang-Nguyen DT Jónsson B Lokoš J. Introduction to the third annual lifelog search challenge (LSC'20). In: Proceedings of the 2020 International Conference on Multimedia Retrieval. Dublin (2020). p. 584–5.

[8] Silva A Pinho S Macedo L Moulin C. Does SenseCam improve general cognitive performance. Am J Prev Med. (2013) 44:302–7. 10.1016/j.amepre.2012.11.005 23415129

[9] Zhou L Hinbarji Z Dang-Nguyen DT Gurrin C. Lifer: an interactive lifelog retrieval system. In: Proceedings of the 2018 ACM Workshop on The Lifelog Search Challenge. Yokohama (2018). p. 9–14.

[10] Li J Zhang M Ma W Liu Y Ma S. A multi-level interactive lifelog search engine with user feedback. In: Proceedings of the Third Annual Workshop on Lifelog Search Challenge. Dublin (2020). p. 29–35.

[11] Duane A Gurrin C Huerst W. Virtual reality lifelog explorer: lifelog search challenge at ACM ICMR (2018). In: Proceedings of the 2018 ACM Workshop on the Lifelog Search Challenge. Yokohama (2018). p. 20–23.

[12] Bilal HSM Khan WA Lee S. Unhealthy dietary behavior based user life-log monitoring for wellness services. In: International Conference on Smart Homes and Health Telematics. (Paris: Springer) (2017). p. 73–84.

[13] Kawamoto K Tanaka T Kuriyama H. Your activity tracker knows when you quit smoking. In: Proceedings of the 2014 ACM International Symposium on Wearable Computers. Seattle, WA (2014). p. 107–10.

[14] Nishiyama Y Okoshi T Yonezawa T Nakazawa J Takashio K Tokuda H. Toward health exercise behavior change for teams using lifelog sharing models. IEEE J Biomed Health Inf. (2015) 20:775–86. 10.1109/JBHI.2015.2478903 26390505

[15] Kumar G Jerbi H Gurrin C O'Mahony MP. Towards activity recommendation from lifelogs. In: Proceedings of the 16th International Conference on Information Integration and Web-Based Applications & Services. Hanoi (2014) p. 87–96.

[16] Uno A Itoh T. MALL: A life log based music recommendation system and portable music player. In: Proceedings of the 29th Annual ACM Symposium on Applied Computing. Gyeongju (2014). p. 939–44.

[17] Nakamura Y Itou T Tezuka H Ishihara T Abe M. Personalized TV-program recommendations based on life log. In: 2010 Digest of Technical Papers International Conference on Consumer Electronics (ICCE). Las Vegas, NV: IEEE (2010). p. 143–4.

[18] Holzinger A. Interactive machine learning for health informatics: when do we need the human-in-the-loop? Brain Inf. (2016) 3:119–31. 10.1007/s40708-016-0042-6 PMC4883171 27747607

[19] Koelstra S Muhl C Soleymani M Lee JS Yazdani A Ebrahimi T. Deap: a database for emotion analysis; using physiological signals. IEEE Trans Affect Comput. (2011) 3:18–31. 10.1109/T-AFFC.2011.15

[20] Samara A Menezes MLR Galway L. Feature extraction for emotion recognition and modelling using neurophysiological data. In: 2016 15th International Conference on Ubiquitous Computing and Communications and 2016 International Symposium on Cyberspace and Security (IUCC-CSS). Granada: IEEE (2016). p. 138–44.

[21] Shin D Shin D Shin D. Development of emotion recognition interface using complex EEG/ECG bio-signal for interactive contents. Multimedia Tools Appl. (2017) 76:11449–70. 10.1007/s11042-016-4203-7

[22] Tripathi S Acharya S Sharma RD Mittal S Bhattacharya S. Using deep and convolutional neural networks for accurate emotion classification on DEAP dataset. In: Twenty-Ninth IAAI Conference. San Francisco, CA (2017).

[23] Ma J Tang H Zheng WL Lu BL. Emotion recognition using multimodal residual LSTM network. In: Proceedings of the 27th ACM International Conference on Multimedia. Nice (2019). p. 176–83.

[24] Lin H Jia J Qiu J Zhang Y Shen G Xie L. Detecting stress based on social interactions in social networks. IEEE Trans Knowl Data Eng. (2017) 29:1820–33. 10.1109/TKDE.2017.2686382

[25] Du J Zhang Y Luo J Jia Y Wei Q Tao C. Extracting psychiatric stressors for suicide from social media using deep learning. BMC Med Inf Decis Making. (2018) 18:77–87. 10.1186/s12911-018-0632-8 PMC6069295 30066665

[26] Kołakowska A Szwoch W Szwoch M. A review of emotion recognition methods based on data acquired via smartphone sensors. Sensors. (2020) 20:6367. 10.3390/s20216367 PMC7664622 33171646

[27] Spathis D Servia-Rodriguez S Farrahi K Mascolo C Rentfrow J. Sequence multi-task learning to forecast mental wellbeing from sparse self-reported data. In: Proceedings of the 25th ACM SIGKDD International Conference on Knowledge Discovery & Data Mining. Anchorage, AK (2019). p. 2886–94.

[28] Lin S Wu X Martinez G Chawla NV. Filling missing values on wearable-sensory time series data. In: Proceedings of the 2020 SIAM International Conference on Data Mining. (Cincinnati, OH: SIAM) (2020). p. 46–54.

[29] Horne J. Why we Sleep. Oxford: Oxford University Press (1992).

[30] Sathyanarayana A Srivastava J Fernandez-Luque L. The science of sweet dreams: predicting sleep efficiency from wearable device data. Computer. (2017) 50:30–38. 10.1109/MC.2017.91

[31] Fishman EI Steeves JA Zipunnikov V Koster A Berrigan D Harris TA. Association between objectively measured physical activity and mortality in NHANES. Med Sci Sports Exerc. (2016) 48:1303. 10.1249/MSS.0000000000000885 26848889 PMC4911242

[32] Soleimaninejadian P Wang Y Tong H Feng Z Zhang M Liu Y. THIR2 at the NTCIR-13 lifelog-2 task: bridging technology and psychology through the lifelog personality, mood and sleep quality. In: Proceedings of the NTCIR-13 Conference. Tokyo (2017). p. 20.

[33] Kato MP Liu Y. Overview of NTCIR-13. In: Proceedings of the NTCIR-13 Conference. Tokyo (2017).

[34] Goldberg LR. An alternative “description of personality”: the big-five factor structure. J Pers Soc Psychol. (1990) 59:1216. 10.1037/0022-3514.59.6.1216 2283588

[35] Wang Y Zhang M Soleimaninejadian P Tong H Feng Z. Big five personality measurement based on lifelog. In: Proceedings of the 2nd Workshop on Lifelogging Tools and Applications. Mountain View, CA (2017). p. 25–8.

[36] McCrae RR Costa PT Jr. A contemplated revision of the NEO Five-Factor Inventory. Pers Individ Diff. (2004) 36:587–96. 10.1016/S0191-8869(03)00118-1

[37] Soleimaninejadian P Zhang M Liu Y Ma S. Mood detection and prediction based on user daily activities. In: 2018 First Asian Conference on Affective Computing and Intelligent Interaction (ACII Asia). Beijing: IEEE (2018). p. 1–6.

[38] Poon JM. Mood: a review of its antecedents and consequences. Int J Organ Theory Behav. (2001) 4:357–88. 10.1108/IJOTB-04-03-04-2001-B008

[39] Roshanaei M Han R Mishra S. Having fun?: Personalized activity-based mood prediction in social media. In: Prediction and Inference from Social Networks and Social Media. Springer (2017). p. 1–18.

[40] Park K Lee S Kim E Park M Park J Cha M. Mood and weather: Feeling the heat? In: *Proceedings of the International AAAI Conference on Web and Social Media. Vol. 7*. Cambridge, MA (2013).

[41] Thayer RE. The Biopsychology of Mood and Arousal. Oxford University Press (1990).

[42] McClung CA. How might circadian rhythms control mood? Let me count the ways. Biol Psychiatry. (2013) 74:242–9. 10.1016/j.biopsych.2013.02.019 23558300 PMC3725187

[43] Beck AT Alford BA. Depression: Causes and Treatment. University of Pennsylvania Press. (2009).

[44] Alghowinem S Goecke R Wagner M Parker G Breakspear M. Eye movement analysis for depression detection. In: 2013 IEEE International Conference on Image Processing. Melbourne, VIC: IEEE (2013). p. 4220–4.

[45] Karp DA. Living with depression: Illness and identity turning points. Qual Health Res. (1994) 4:6–30. 10.1177/104973239400400102

[46] Hirschfeld RM Klerman GL Clayton PJ Keller MB. Personality and depression: Empirical findings. Arch Gen Psychiatry. (1983) 40:993–8. 10.1001/archpsyc.1983.01790080075010 6615162

